# Health Policy for Persons with Intellectual Disability: Experiences from Israel

**DOI:** 10.1100/tsw.2005.14

**Published:** 2005-01-28

**Authors:** Ilana Halperin, Aliza Shupac, Mohammed Morad, Joav Merrick

**Affiliations:** ^1^ Schulich School of Medicine, Faculty of Medicine and Dentistry, University of Western Ontario, Canada; ^2^ Faculty of Arts, Department of Political Sciences, McGill University, Montreal, Quebec, Canada; ^3^ Clalit Health Services and Division of Community Health, Ben Gurion University of the Negev, Beer-Sheva, Israel; ^4^ National Institute of Child Health and Human Development, Ministry of Social Affairs, Jerusalem and Zusman Child Development Center, Division of Pediatrics and Community Health, Ben Gurion University of the Negev, Beer-Sheva, Israel

**Keywords:** intellectual disability, developmental disability, mental retardation, health policy, public health, Israel

## Abstract

Intellectual disability (ID) is a life-long disability characterized by impaired cognitive and adaptive skills. Over the past few decades, a shift has occurred in the conceptualization and treatment of people with ID and research in health policy and health-care delivery has become increasingly global with a notable disparity between the developed and developing world. This review presents a literature overview of global health policy for ID with the intent to focus specifically on the policy and treatment within Israel. The methodology involved sites visits to care centers, discussions with stakeholders in health policy, and a literature review. We believe that Israel is in a unique position between a developed and developing culture. In particular, the distinct problems faced by the Arab and Bedouin community in terms of ID must be formally accounted for in Israel's future policies. Research from the developing world would be instructive to this end. The global approach in this presentation led to certain policy recommendations that take into account the uniqueness of Israel's position from a social, economic, religious, and demographic perspective. It is the hope that this paper will lead to an increased awareness of the challenges faced by persons with ID and their providers in all sectors of Israeli society and that the necessary policy recommendations will ultimately be adopted.

### INTRODUCTION

Intellectual disability (ID), mental retardation (MR), or developmental disability (DD) refer to a life-long disability characterized by impaired cognitive and adaptive skills. The past few decades have witnessed a shift in the conceptualization and treatment of persons with ID. Research in the area of health policy and health-care delivery for people with ID has become increasingly global with a notable disparity between the developed and developing world. This paper presents a literature review of global health policy for ID. It is the intent of this paper to focus specifically on the policy and treatment of this population within Israel with attention to the country's political and social subsystem. This discussion is then contextualized within the global system. The paper argues that in terms of health policy and health-care delivery for persons with ID, Israel falls somewhere between the developed and the developing world. As a result, its unique position, lessons learned, and research conducted can be used to help further policy in developing countries. Likewise, Israeli policy makers should look to the experiences of the developed world in order to create a succinct health policy for the intellectually disabled. The methodology employed for this study involved site visits to residential and community care centers; discussions with stakeholders in health policy for persons with ID including parents, guardians, physicians, social workers, nurses, physiotherapists, occupational therapists, speech language therapists, and policy makers; as well as a literature review of global and Israeli health policy and research.

### ISRAEL IN THE GLOBAL PICTURE

Israel is unique in that it has qualities of both a developed and developing country. Israel's booming high-tech industry, flourishing academic centers, and contributions to global research point to a developed society. In contrast, aspects of society such as the increasing poverty rates, the growing gap between upper and lower classes, the security risks, and the difficulty in service provision for the growing Arab minority are indicators of a developing country.

Social problems faced by Israel are similar to those facing other Western countries. They are often interrelated and analogous. In light of the recent origins of the State of Israel, the striking similarities between Israel and the developed world can be viewed as an impressive feat for Israel. It is also impressive considering the soaring number of recent immigrants to Israel (1,130,000 in the past 14 years, mostly from the former Soviet Union[1]) that must be absorbed in conjunction with a flailing economy and high security threats. Despite all of these pressing issues, Israel has adopted a progressive stance on issues facing people with ID and provides a number of innovative services for this population. Community housing developments have begun to proliferate since the 1980s and a shift to a more normative and community-oriented approach has been in place since the 1960s[2]. The majority of the challenges confronting Israel right now, in the realm of ID, are a result of gaps in health service delivery that essentially mirror the gaps in the rest of the developed world. Despite the parallels between Israel and the West, there are a number of problems in terms of health service delivery for Israel that are more analogous to the developing world, particularly in the realm of services for the Israeli-Arab population.

As of 2003, the population of Israel stood at 6,727,000, close to 80% Jewish and the remaining minority predominately Arab[3]. Although the country was originally founded on social democratic principals, the present government's fiscal policy has dictated a large downscaling of the state's share in the national economy. A recent report conducted by the Center for Social Policy Studies in Israel (CSPS) indicated that unemployment, income-maintenance benefits, education, and health expenditure declined in recent years[4]. The annual CSPS Public Opinion Survey showed that the public felt the implications of the economic crisis acutely this year. Most respondents believed that the level of social services had declined, socioeconomic gaps had widened, and the standard of living had fallen[4]. More than 40% of respondents noted that they felt very vulnerable to the possibility that unemployment would affect them directly[4].

Poverty in Israel has increased in the last decade, from 13% of families living below the poverty line in 1989 to 19% in 2003[3]. The incidence among families with children rose from 17.9% in 1998 to 32.7% in 2000. As of 2000, it was found that 25% of children in Israel lived in poverty[5]. When compared with other developed countries, only the U.S. had worse poverty incidence than Israel[5]. Socioeconomic status and education were strong predictors of health and there was a consistent ranking order in Israeli with Ashkenazi Jews, followed by Mizrahi Jews, and then Arab Israelis[6].

In Israel, antenatal care, geriatric, and mental health services are provided by the Ministry of Health, while curative services are dispensed by four not-for-profit health insurance providers[6]. On January 1, 1995, the National Health Insurance Law went into effect[6]. The law set forth the state's responsibility to provide health services for all residents of the country. The law stipulated that a standardized basket of medical services, including hospitalization, would continue to be supplied by the sick funds. Every resident must register as a member with one of the four sick funds. The sick funds may not bar applicants on any grounds including age or state of health. Sources for funding of health costs include progressive health insurance premiums paid by each resident, employers' health tax payments, National Insurance Institute funds, funds from the Ministry of Health budget, and consumer participation payments[6]. As with other social programs in recent years, there has been a decline in governmental spending in health care. As of 2003, the national expenditure on health as a percent of GDP stood at 8%, which was lower than the majority of developed countries. Some 20% of the population had refrained at least once in the past year from using a medical service due to its cost. Households finance one-third of national health expenditure today, as against about one-fourth in the mid-1990s[4].

### INTELLECTUAL DISABILITY

#### Shifts in the Policy and the Definition

The developed world has undergone a shift in ideology and practice concerning health policy in the past 40 years that has led to profound changes in the treatment and perception of people with ID[7].

The shift included the process of deinstitutionalization, as institutions closed and their former inhabitants and would-be inhabitants remained instead within the community setting. Thus, the shift can be viewed as a transition to a more community-oriented approach. This is apparent in a review from Scotland[8] that advocated that "but for all but a few people, health and social care should be provided in their own homes or in community settings alongside the rest of the population." This recommendation conforms to today's reality in Scotland as a majority of persons with ID are cared for in the home. Statistics indicated that in 1980, 6,500 persons with ID lived in institutions, whereas the number was 2,450 in 1998[8].

The community programs that were developed from the late 1960s onwards were a result of the social and ideological trends of the early 1960s in Scandinavia and the U.S., where family members of persons with ID began to advocate for complete social inclusion[9]. This shift towards normalization and societal inclusion soon spread to other parts of the developed world, as indicated by the 1971 United Nations Declaration of Human Rights for individuals with mental retardation[9]. The goal of this movement was to ensure that the lives of people with ID should be as normal as possible[9]. The process in the developed world first focused on physical integration within the community followed by the self-advocacy movement, which witnessed apartments and community homes supplanting institutions and supported employment supplanting sheltered workshops[9].

The process of deinstitutionalization brought with it a number of important benefits in the form of newly established rights for individuals with disabilities. These included the right to public education in the least restrictive environment and the right to publicly funded health-care coverage. The group homes that came to supplant institutions generally exhibit improved staff-to-resident ratios[7].

A further, albeit interrelated, shift occurred on an ideological and policy framework level. This shift witnessed the "death of the medical model" and the subsequent birth of the community health supports model[10]. Like the aforementioned shift, it is based on the belief that normalization of persons with ID should occur so they can be integrated as fully as possible into the community at large. It encompasses an entirely novel way of conceptualizing health with substantial implications for the realm of health policy and health delivery for persons with ID. The medical model involved the “medicalization of disability”, where the problem was viewed as part of the individual who was believed to be abnormal, sick, diseased, or inferior instead of looking at the actual functioning and positive abilities of that person within the environment or society. That model supported unequal roles between physician and patient. The physician was regarded as the possessor of all relevant knowledge and consequently given control over the patient’s health at the expense of his or her personal input. As a result of this unequal relationship, treatment under this model was provided in hospital-type settings with a lack of familial or individual control[10]. The new community health supports model is based on the World Health Organization’s (WHO) definition of health, “A state of complete physical, mental and social well-being”[10]. This view is also reflected in the international classification of functioning (ICF), as a health condition (disease or disorder) that impacts personal functioning through[10]:

1. Problems of body structures and functions (impairments)
2. Difficulties in performing desired (self-chosen) activities (limitations)
3. Participations in preferred life situations (restrictions)
4. Modified by contextual factors

The 2002 AAMR (American Association for Mental Retardation)[11] definition of mental retardation is reflective of this shift. It defines mental retardation as: “A disability characterized by significant limitations both in intellectual functioning and in adaptive behavior as expressed in conceptual, social and practical adaptive skills. This disability originates before age 18 years.” This definition focuses on the level of functioning of individuals, “their adaptive behaviour as expressed in conceptual, social, and practical skills”[11] and their performance capabilities whereas earlier definitions focused on IQ as a measurement of mental retardation. A comparison of the 2002 system’s dimensions with that of the 1992 system’s dimensions is quite revealing to this end[11]:

**The 1992 System:**
Dimension I: Intellectual Functioning and Adaptive Skills
Dimension II: Psychological and Emotional Considerations
Dimension III: Health and Physical Considerations
Dimension IV: Environmental Considerations

**The 2002 System:**
Dimension I: Intellectual Abilities
Dimension II: Adaptive Behavior (conceptual, social, practical skills)
Dimension III. Participation, Interactions, and Social Roles
Dimension IV. Health (physical health, mental health, etiology)
Dimension V: Context (environments, culture)

The community supports model, reflective of the new terminology, advocates self-determination on the part of the person in the determinants of health. Instead of advocating for the medicalization of ID as earlier, it emphasizes the provision of supports to enhance individual levels of functioning. Instead of advocating hospital settings as the former model does, it advocates the full participation of ID individuals in community life[10].

The patient-physician roles are viewed as equal, treatment is viewed as equal, and community-based settings are advocated[12]. This new conception of health advocates a certain, needed directional shift in terms of health delivery and policy, but it also reflects the shift towards normalization and deinstitutionalization with its accompanying ideological shifts that began approximately 40 years ago.

Moreover, it provides a benchmark with which to measure the progress in health services and health policy and “offers a vision of what health should be in the future”[10].

While the aforementioned shifts can ultimately be viewed as positive for their progressive stances on people with ID, the transition from institutionalization and the medical model to a community supports model is not without accompanying problems. The deinstitutionalization process, while arguably leading to a more compassionate and supportive way of living for people with ID, has not been accompanied by necessary changes in the provision of health services to this population. An unfortunate by-product of the transition was a widening gap between health service delivery and health needs. Moreover, like the institutions that preceded them, current health service delivery for ID persons is characterized by severe financial shortages[12]. Public attitude has begun to change since the process of deinstitutionalization began, but more change is needed in order to overcome negative attitudes about the inclusion of persons with ID within the community, particularly within the work environment and in terms of receiving equal medical and service provisions[7].

The attitudes of community members, support workers, and members of the academic and medical community are vital to ensure that the principles of community living are carried out in practice. The quality of life for persons with ID is, in large part, contingent on the way that their fellow community members perceive them. For example, public opinion influences public policy decisions and funding priorities[9]. In particular, the attitudes of support workers in community programs can play a large role in determining the quality of life of persons with ID[9]. The importance of workers’ attitudes is even more significant in community agencies than in institutional settings. For in the latter, staff are closely supervised and, thus, can still be ensured to follow policy guidelines. In the former setting, however, the workers are not closely supervised, but must truly be committed to helping persons with ID to live independently within the community in order for the goals of community living (particularly inclusion and empowerment) to be met[9]. This is enhanced by the decentralized nature of most agencies[9]. Recent studies have generated concern about the commitment of staff workers to further such goals and their ability to implement inclusion philosophy[10]. This is highly problematic as the staff workers are the most important agents in the creation of a community and inclusive approach for the patients, as they are the ones who are able to implement agency values and “it is they who determine the extent to which empowered community-living will become a reality for those they serve”[9].

Studies have raised concern that staff workers do not exhibit more positive attitudes towards persons with ID than the general community and one study even found that community support staff members had less positive attitudes than the comparison group[9]. Thus, despite the shift to a community approach and deinstitutionalization, there is a time lag between this philosophical approach and the attitudes of staff members who are charged with implementing it[9].

A multinational study of attitudes toward individuals with ID, which studied attitudes in ten countries (Brazil, China, Egypt, Germany, Japan, Nigeria, Republic of Ireland, Russia, UK [Northern Ireland], and the U.S.), concluded that “inclusion, at least as a philosophy, has become broadly accepted, the full acceptance of individuals with ID has (however) not yet been achieved”[13]. In most of the countries surveyed, a majority of people believed that the inclusion of persons with ID into the workplace and schools would “create more accidents on the job, cause discipline problems in the classroom, lower productivity and negatively affect the learning of other students”[13]. Thus, a gap could be seen in terms of the formal adoption of a more community-oriented approach and the attitudes held by most members of society. There is also a general perception that people with ID are not lacking in health care[13]. This too is problematic for the attitudes held by the general community are important in terms of ensuring policy formulation. If there is no perceived problem in this area by the general public, then it is likely that no policy changes will be made despite studies that indicate the importance of doing so. Throughout the world, the family is regarded as the main caregiver, in part because of culture and in part because of a lack of resources[13].

### Israeli Policy

In Israel, the Division for Mental Retardation (DMR), under the auspices of the Ministry of Social Affairs, provides services to people with ID. This service is provided to a total of about 25,000 persons with a diagnosed ID[2]. Of those, about 8,000 are living out of home, 6,000 in 58 residential care centers around the country, and 2,000 in community-based hostels and protected apartments[2,14]. About 17,000 are served with day-care kindergarten, day-treatment centers, sheltered workshops, or integrated community care[2,14]. In 1999, the DMR expenditure totaled US$793 per person served per month[15]. The government covers the cost of health care for those living in residential care centers, which includes 24-h nursing staff and frequent physician visits[2]. The health care of those living in the community is administered through the four sick funds. At present, this entitles people with ID to the same basket of services that the general population receives, however, it is the position of this paper that those living in the community require more comprehensive care than the sick funds can provide. It is incumbent on the DMR and the Ministry of Health to cooperate in addressing the unique health-care needs and current barriers to access of this population.

The intellectually disabled population in Israel is covered under the laws addressing all disabilities; these include the “Guardianship Law” of 1962, “Residential Care Center Law” of 1965, the “Welfare Law for Persons of ID” of 1969, the “Minority Protection Law” of 1989, “Special Education Law” of 1988 (amended in 2002), and the “Equal Rights for People with Disabilities Law” of 1998[16]. The only legislation that specifically addressed health care in this population is the inclusion of Child Development Services in the National Health Insurance Law of 1995[16]. In the 1970s, clear policy was developed regarding criminal acts, mandatory education, and marriage in the ID community[2]. At the same time, a medical director was appointed in the DMR and a chief nurse position followed soon after[2]. In the 1980s and 1990s, residential care centers were expanded, waiting lists were abolished, community services expanded, and parents were encouraged to keep their children at home as long as possible[2]. A 2003 assessment of the medical services for ID persons in Israel concluded that “within the framework of Division of Mental Retardation of the Ministry of Social Affairs medical service is provided at an acceptable level, even with the lack of adequate manpower, but within the community there is a lack of relevant services”[2]. Those persons with ID living in residential care facilities have their health-care needs adequately addressed, but those in the community are at a disadvantage. According to their website, the DMR “acts to improve functioning of the mentally disabled and encourage families and communities to care for them in their natural surroundings”[17]. Thus, the current health service delivery for persons with ID is in contradiction with the mandate of DMR. The developed world trend towards community and in-home services has reached Israel as well, however, the policy regarding the health needs of this unique population lags behind the normalization and deinstitionalization.

The deinstitutionalization movement in Israel was not as influential on government policy as it was in other developed countries such as Scandinavia and the U.S.[2,9]. The first community house in Israel was founded in 1969, but the more significant shift to community living did not take place until the 1980s when more than 90% of the community living agencies opened[9]. Different organizations created different models of community living[9]. A further difference between Israel, Scandinavia, and the U.S. is that in the former, the proliferation of community-living programs “did not affect the existing institutions, which remained as the major alternative for out-of-home placement”[9].

The birth of community-living programs in Israel was sparked when the “father” of normalization and deinstitutionalization, Niels Erik Bank-Mikkelsen from Denmark, came to Israel for a visit in 1976 and published a report in which he recommended “improving services by increasing the role of municipalities to this end, developing alternatives to residential treatment, emphasizing the professionalization of home workers, increasing the number of workers per resident and raising the status of the workers”[2,9]. The publishing of his report initiated a process of reorganization that led to the development of an organizational infrastructure to ensure a more efficient administration and greater integration between service and parent organizations[9]. This process was bolstered by the adoption of a community-oriented approach. The effects of this shift can be seen in the 1993 policy of the Ministry of Social Affairs concerning the waiting list for residential homes and giving parents the ability to choose between various out-of-home living facilities for their child[9]. Parents were encouraged to choose facilities and treatment plans situated within the community with the municipalities taking responsibility for community housing, placement, and diagnosis[9]. By 1999, 1,150 persons with ID were living in community housing, up from 598 persons in 1993[9]. Local public and nonprofit parents' organizations, forming a loose amalgamation of interests groups without any "global policy or structure", primarily organized the shift to community housing. A total of 75% of community living was provided by public organizations, while only 25% was provided by the private sector[9]. The goal of such programs was to ensure that small programs existed to provide the maximum amount of community inclusion and participation and a high quality of life for participants[9].

Services for the disabled are provided by the government, the National Insurance Institute, the Commission for Equal Rights for People with Disabilities, the health funds, local authorities, voluntary organizations, and the private sector[16]. The number of children who need services is two to three times greater than the number of children actually receiving services. The gaps between needs and provided services are particularly large in the Arab sector and in the periphery (north and south)[18]. For example, in 2000, a new law mandated the establishment of rehabilitative day-care facilities for children with disabilities under the age of three. This law is only partially implemented on a small number of children (about 250 out of 3,000) who are in need of this service[19].

There are a number of small not-for-profit organizations that provide community living options for persons with ID. AKIM (National Association for the Rehabilitation of the Mentally Handicapped in Israel) represents 30,000 persons with ID and their families. AKIM services include day-care centers for infants and hostels, apartments, and employment opportunities for adults[20]. AKIM also functions as a lobby group that pressures the courts to improve legislation for the ID community. The dual role of lobby group and service provider is seen by some as a conflict of interest and may weaken their position in the eyes of the court. AKIM does advocate for the ID community, but it treads carefully around the policy makers because it relies heavily on governmental funds. Another organization, Aleh, provides a "stimulating and nurturing environment" to 500 children. The services include 24-h medical and nursing care, nutritionist-planned diets, special transportation, hydrotherapy, occupational therapy, physiotherapy, speech therapy, music therapy, sensory stimulation, and art therapy[21]. SHEKEL, another larger service for disabled people, provides assessment and treatment, education, community living, and vocational rehabilitation. Foundations and fees for services compromise 20–25% of SHEKEL's operating budget, while government and local authorities cover the remainder[22]. These organizations are important providers of social and rehabilitation services. Interestingly, none state basic health care as a policy concern.

Around the Beer-Sheva area in the south of Israel, where the two first authors stayed as summer interns in 2004, there are a few examples of community living for the intellectually disabled. The Beit Cohen center is a community housing complex dedicated to adults with ID. It is funded through the assistance of private donations, Ministry of Social Affairs, National Insurance, and the Municipality of Beer-Sheva. It is fully supervised by the Ministry of Social Affairs and the Municipal Social Services. The residents are involved in community and city life. The residents work during the day in protected or sheltered workshops and have a routine afternoon where they participate in leisure and social activities[23]. The residents receive their health care through the general sick fund, but there has been no research to indicate if this health care is sufficient.

Another unique community residence is Kfar Rafael, just outside Beer-Sheva. Kfar Rafael, a rehabilitation community, extends over an area of about 120,000 $m^2$, 40,000 of which are designated for residences and 80,000 for agriculture. The current population counts approximately 110, of whom 50 are villagers who have ID, 7 foster families, as well as 20 volunteers. Each couple heads an enlarged family, consisting of their own children, two staff members, and six villagers. The family provides a home with a warm, receptive atmosphere, free from the pressures of work or studies. The couple's children interact freely with the villagers. Kfar Rafael is operated as a not-for-profit organization, funded and supervised by the Ministry of Social Affairs. All of the villagers are placed through the DMR[24]. The therapeutic philosophy is based in anthroposophic medicine; chief among these teachings is that the body is not an independent material organism and that good health depends on a harmonious relationship between the physical body, spirit or vital force, the soul, and the ego[25]. In Kfar Rafael, every resident contributes according to his or her own potential. There are some special health concerns for an institution such as Kfar Rafael and other community dwellings where there is little to no physician involvement. For example, Kfar Rafael is strictly vegetarian and for many years, the residents there were vitamin B12 deficient[26]. In addition, a higher incidence of *Helocobacter pylori* was found in this specific institution, both among the persons with ID and the volunteers[27]. Without properly targeted medical attention, these unique ailments of community dwelling can go unnoticed. Physicians dealing with these populations must receive the appropriate training and knowledge. It appears from these examples that the ID community is being forced to sacrifice the health care they deserve in exchange for a place in the community.

### HEALTH CARE FOR PERSONS WITH INTELLECTUAL DISABILITY IN THE DEVELOPED WORLD

#### Caregivers and Costs

The expenditures for persons with ID are costly and time consuming and can be emotionally and financially draining for caregivers. Also, the shift to a community approach and the deinstitutionalization process maintains that the individual should be at the center of policy development. As such, flexibility in general and more specifically in terms of financing service delivery is essential to developing the best service provision. The “Same as You: Learning Disability Review”[8] advocated for local area coordinators to provide direct payment of funding to the customer so that they can elect to pay for whatever services they personally need instead of using services provided by an agency financed by a block grant[8]. Some caregivers or persons with ID may not wish to take on this task, perceiving it as an extra burden, in which case the local coordinator or service providers should provide services accordingly[8]. Funding for services is vital to ensure that persons with ID have their needs met as people with ID are overrepresented in the poor segment of society and live on government benefits[12].

#### Lack of Support for Families and Primary Caregivers

The family has become the primary caregiver for persons with ID. One of the major obstacles to providing appropriate care for persons with ID and their caregivers is the accessibility of relevant and coordinated information. Professionals often lack the relevant information or the time to help the families learn of existing services, of their rights, and their ability to access such services. Informal information exchange between other ID family caregivers has been cited as one of the main methods of learning about such services. The absence of coordinated information heightens the stress and burden for caregivers. Thus, there is a need for a more coordinated and organized approach to supplying caregivers with such information. There is a need for organizations to work with individuals and their families to discover, which services are available to them[12]. More effective communication and referral pathways are needed between health-care professionals[8].

#### Medical Concerns

Persons with ID have a higher prevalence of medical conditions such as epilepsy, dementia, thyroid problems, osteoporosis, sight problems, hearing problems, underweight or overweight, and mental health problems[8,12,28]. Often, these medical problems go undetected or are insufficiently treated as a result of difficult or lack of communication between patient and care provider. The result is that individuals with ID generally have poorer health and shorter lifespans[8,12,28].

### Oral Hygiene

Ensuring proper oral hygiene and dental care for persons with ID requires a specialized form of dentistry sometimes referred to as “special care dentistry” in order to deal with the needs of this special population. This generally involves altering the dental examination and dental treatment according to the disability of the patient. Generally, more coordination between the dentist and other health-care providers is required, as well as taking time to accommodate the individual as well as his or her caregivers, providing more time for individual patients, communicating through an interpreter, and planning a treatment with the patient to account for potential future dental concerns. Oral hygiene problems pertaining particularly to ID persons may stem from the difficulty to enforce proper personal dental care such as adequate toothbrushing. Also, treatment may differ because communication problems may lead to a later onset of dental problems. Broken teeth may be more common due to poor ambulatory skills, seizure disorders, or chewing on nonedible objects Additional problems include insufficient numbers of dental health providers or trained dental health providers for people with disabilities These concerns will become increasingly problematic as people with ID are now living longer. Inadequate financial coverage and lack of reimbursement for dental services clearly prevent properly addressing the dental needs of individuals with special needs[12,28,29].

### Barriers to Care

People with ID today are living longer than ever before and with this aging process comes many health concerns not addressed by previous generations of physicians. The difficulty in treating this population is due to communication and behavioral barriers. In community settings where physicians lack the training, the time, and the financial incentives, this often results in medical problems often ignored, delayed, or untreated[2,28]. As mentioned, communication problems place an added barrier to ensuring that these people are properly treated[12], while other barriers include lack of training for physicians pertaining to ID and its related health issues, lack of effective screening of the population, and lack of full medical history of the individual[30]. Physicians also express difficulty with patient compliance, consultation time limitations, poor continuity of care, and inadequate knowledge of services and resources[31].

### Grandparents Raising Grandchildren with Developmental Disabilities

In order to provide adequate provision of health services, the phenomenon of grandparents raising children with ID should be acknowledged. Research has shown that grandparents in such caregiving roles may suffer from stress, depression, and ambiguity concerning their roles as grandparents. There is a need for more planning for this particular subset of the caregiving community, especially as demographic trends point to a growing increase in grandparent-headed families in every socioeconomic and ethnic segment. Some of the particular problems for this population are the higher levels of health problems for the grandparent caregivers themselves and finding adequate housing, as policies have not addressed the special needs of the grandparent caregiver. Because grandparents in senior housing or other public housing could face eviction should their grandchildren live with them, there is a need to gather more data on this group to ensure that their needs are adequately addressed[32]

### Double Disadvantage

It is generally acknowledged that people with learning disabilities who are from an ethnic minority are at an even greater disadvantage than those individuals with ID who are part of the mainstream culture and ethnic background[12]. Another doubly disadvantaged subsection of the ID population is women. People with ID are living longer today than ever before and women generally outlive their male counterparts. Health professionals must take into account that women with Down syndrome are likely to experience sensory, adaptive, or cognitive losses earlier than most other women. Furthermore, women in general have certain reproductive issues ranging from puberty to menopause that the medical community must take into consideration. Women with ID need special help and additional time in order to cope with these physical changes[33].

## HEALTH CARE FOR PERSONS WITH INTELLECTUAL DISABILITY IN ISRAEL

### Aging Population

A recent study in Israel that surveyed the health status and services use of older adults with ID living in residential care centers had similar findings to a large-scale, population-based survey of health status and health services use among older people with ID living in community-based small group homes in Upper New York state[34,35]. Both studies indicated that older persons with ID age very much like other adults and in general experience the same health problems, albeit with some variations in frequency and disability-related conditions. Both studies noted that underreporting of risk factors may occur among persons with ID for many reasons including compromised ability of adults to report illness or uneven application of aggressive primary prevention strategies and practices such as exercise programs and dietary management[34,35].

### Educating Health-Care Workers

In a study of the inclusion-related attitudes of community agency staff in the U.S. (147 staff members) and Israel (74 staff member from all areas of Israel), it was found that higher educational levels, regardless of nation, were associated with higher empowerment scores (on the Community Living Attitudes Scale, CLAS), lower exclusion scores, lower sheltering scores, and higher similarity scores[9]. Most of the staff in Israel were women under the age of 34 years (46%) with either full or partial academic degrees (65.8%). A most disturbing finding by the study was that training in inclusion philosophy did not lead to more positive inclusion attitudes. However, studies conducted in the U.S. have shown that proper training can indeed foster attitudes that advocate community inclusion and individual empowerment[9]. If the issue is inclusion, then this study showed that there is a need for training Israeli staff members in this concept and, through the recruiting process, find staff workers who believe in inclusion philosophy[9]. These findings suggest that further research should be performed to motivate staff members to pursue the principles of community living for persons with ID[9]. The negative attitudes espoused by Israeli support staff is largely on par with the attitudes that pervade the Western world in terms of perception and treatment of persons with ID[13].

In the year 2000, a group of concerned physicians and allied health-care professionals formed an ID interest group to discuss the health issues in the ID community. Together with the School of Continuing Medical Education at the Ben Gurion University and the Department of Family Medicine, several small seminars on ID were conducted for the primary care physician. In the year 2002, a two-semester course on disability was initiated for allied professions at the Recanati School for Community Health Professions in Beer-Sheva with the participation of 25 students each year. These initiatives come from the bottom up, from a group of concerned health-care workers who saw a void and attempted to fill it. These projects remain fragmented and it will take a long time to reach a wider service-provider population. Without the proper policy and resource allocation from the top down, the issue of health education and ID will not be rectified soon.

### Special Health Concerns for the Person with ID in the Community

One study in Jerusalem was conducted to compare the aging phenomena of people with ID, aged 40 years and above, living in community residences to those living at home with their families[29]. The purposes of the study were to compare the health status between the two types of settings, to compare the health status between the sample and the general Israeli population of the same age group, and to investigate whether deterioration occurred in Activity of Daily Living (ADL), cognitive ability, and leisure activity. Health problems had already appeared by age 40 among the participants. The most frequent problems were visual impairment (33%), hearing impairments (20%), and heart problems (20%). Dental problems were found in 30%. The community-based residence group displayed more medical problems than people living at home, whereas individuals living at home had more dental problems. The functioning of the participants in the ADL areas was high, with no evident decline reported during the previous 5 years. Concerning leisure time, a decline in functioning in both residential groups was observed and interestingly, the scores for social life and leisure activities were better for the community-based residential group.

One aspect of health that was considerably different between the community and home dwellers was dental hygiene. Dental care is less available to the population with ID living at home[29]. A wide range of barriers restricts access to dental care. Examples include availability of dentists willing or able to treat this population, lack of training for people with ID as to how to behave at the dentist, how to demonstrate and control pain, and general difficulties in treatment due to behavior and communication problems. In Israel, the DMR provides people with ID living in residential care with free dental care, while those living at home are left to make their own dental care arrangements, as is also the case with the general population. This study resulted in a shift in policy and today the dental clinic in residential care centers around Israel will also provide service for people with ID living at home.

A major health concern for those with ID is sensory impairment. In the study of the aging ID population in Israel, the most prevalent problem was sensory: 33% showed visual impairments and 20% showed hearing impairments[29]. A recent study of two residential care centers in the Negev indicated that this population was not receiving adequate ophthalmologic and optometric care. The patients with vision problems reported difficult viewing television and cooking. The number of participants who had or believed they needed glasses was equal to the number found in need of correction after eye exam[36]. Visual impairment is a debilitating condition that can affect many activities of daily living. This study[36] points to the need for improved service delivery to this group concerning screening for visual impairment.

## HEALTH CARE FOR PERSONS WITH INTELLECTUAL DISABILITY

### The Developing World

Despite the fact that the majority of the world’s children live in the developing world, where these children are at increased risk for ID, there is a dearth of data on both the prevalence and causes of ID. This lack of research can be attributed to both a difficulty in surveying a population where most children with disabilities are not registered formally and the fact that childhood disability is of low priority when childhood mortality rates are still very high[37]. Thankfully, the last decade has seen a decline in infant mortality rates by 1.2%/year[38] and as basic health care improves for the children of the developing world, the life expectancy of children with developmental disabilities will increase. With the increased prevalence of developmental disabilities comes an increased public health need that can no longer be ignored.

The prevalence of severe ID in developing countries is consistently found to be above 5/1,000. While in developed countries, it is less than 5/1,000[37]. As with developed countries, mild ID is found to be more prevalent and is highly correlated to socioeconomic status within a developing country[39].

The causes of developmental disabilities include genetic factors, nutritional deficiencies, exposure to toxins, perinatal and neonatal complications, trauma, and poverty. These different factors, often working in concert, affect the developing nervous system. Although these factors are causes of ID in both the developed and developing world, the higher rates of poverty, poor access to health care, cultural norms, and badly administered or nonexistent prevention and social assistance programs all contribute to the higher rates of developmental disabilities in the developing world.

### Genetic Factors

Due to the high rates of infection and nutritional disorders in the developing world, genetic causes of developmental disability are often given little consideration. Gene mutations can be rapidly transmitted and increased to high frequency via genetic drift within social, religious, and geographical isolates of this type, especially in communities that are numerically small. Due to the restricted nature of their gene pools, there also is a high probability that by chance alone, couples who marry are biological relatives[40]. In many developing countries, there is also a strong cultural preference for consanguineous marriage and so in North and sub-Saharan Africa, the Middle East, West, Central and South Asia, 20% to over 50% of marital unions are intrafamilial, most commonly between first cousins[41]. Consanguinity increases the rate of expression of deleterious recessive mutations in disorders such as phenylketoneuria (PKU), Smith-Lemli-Opitz syndrome (SLO), Prader-Willi syndrome, Angelman syndromes, L1 (a neural cell adhesion molecule)–associated conditions, and autosomal recessive microcephaly[42,43,44]. Due to the small capacity for prenatal and newborn genetic testing in these populations, little research has been done on the specific genetic causes of developmental disabilities in developing countries. In developed countries, it is documented that ID is more prevalent among males than females because a proportion of ID has an X-linked etiology, most commonly fragile X syndrome[43,44]. In countries lacking access to contraception, family planning, and prenatal screening, the number of births to women over the age of 35 years has increased. Thus, the prevalence of Down syndrome may be three times higher than in countries with family planning and genetic screening programs in place[45].

With the increasing contribution of genomics and proteomics to health studies comes a new multidisciplinary approach to genetics in medicine. Community Genetics starts from a medical genetics/community medicine perspective and seeks to provide guidelines for the establishment and surveillance of programs to prevent and control the adverse effects of human genetic disorders[46]. These programs can be run at local, national, and regional levels since they emphasize strengthening the role of primary health care, integrating interventions into reproductive health programs, and ensuring the feasibility and cost effectiveness of preventive strategies[47]. Community Genetics has tended to concentrate on providing services to populations where genetic disorders are present at high frequency and on establishing community-specific care programs. This includes communities in developing countries[40]. There is an important place for community genetics in developing countries, however, the prevention and counseling must occur in a culturally sensitive, nondirective manner. Not every consanguineous marriage results in genetic or congenital disorders and it is important to recognize the cultural traditions that encourage these marriage rituals.

### Nutritional Deficiencies

One of the leading causes of preventable ID is iodine deficiency. *In utero* exposure to maternal iodine deficiency during the first two trimesters of pregnancy can damage the developing brain, causing permanent cognitive disability as well as early-onset motor, hearing, and speech disabilities[37]. Iodine deficiency is a minor concern in developed countries due to the widespread use of iodized salt. Still, more than one-third of the world population, mostly in Asia, is not using iodized salt and is therefore not protected against iodine deficiency disorders[48]. There is a grassroots effort to improve salt fortification programs in those countries not currently protected from iodine deficiency disorders. The International Council for the Control of Iodine Deficiency Disorders was formed in 1985 with support from UNICEF, WHO, and the Australian government to bridge the gap between available knowledge and its application to attain iodine sufficiency[49]. One major issue this group struggles with is education regarding iodine deficiency; many people associate goiter with lack of iodine, but have difficulty relating the deficiency to fetal brain development, as it is not a visible ailment. The United Nations pledged to have iodine deficiency eliminated by 2005, but the recent progress reports from many developing countries indicate that although progress is considerable, it varies widely and much work remains to reach the goal of worldwide iodine sufficiency. In addition, some countries are backsliding, indicating the need for sustainable public communication and education[48].

Other micronutrient deficiencies that are implicated in ID include iron and folate. In childhood, iron deficiency is associated with deficits in cognition, attention, and learning[37]. There is some research to suggest that iron deficiency may also have a direct effect on the developing nervous system during infancy[50]. Folate deficiency preconceptually and in early pregnancy is well documented as a cause of neural tube defects such as spina bifida that can result in both motor and intellectual impairments. Periconceptional supplementation of 400 mcg of folic acid per day is sufficient to prevent the occurrence of more than half of neural tube defects[51]. However, vitamin supplementation in developing countries is still a public health challenge.

### Infection

There is a number of prenatal, postnatal, and childhood infections that can damage the developing nervous system and cause long-term disabilities in children. Many of these are preventable by immunization including rubella, meningitis, and polio. Other infections can be treated with antibiotics or antiretroviral drugs in order to reduce transmission to the fetus such as syphilis and HIV, respectively. Because of the preventable or treatable nature of these infections, they are a minor concern as a cause of ID in developed countries. Despite massive international efforts to promote immunization in the developing world, these infections are still highly prevalent in many developing countries and are thus a common cause of ID[52].

### Toxins

Heavy alcohol abuse in pregnancy is associated with fetal alcohol syndrome, which includes cognitive disability; microcephaly, and facial abnormalities[37]. Childhood exposure to lead has been shown to be associated with fatal encephalopathy, cognitive delay, and hyperactivity[53]. In low-income countries, children continue to be exposed to lead from gasoline[54]. *In utero* exposure to teratogens and early childhood exposure to other toxins has been well documented in the developed world and preventative and education programs implemented. Unfortunately, similar programs are scarce and lacking in developing countries and the magnitude and effects of teratogenic and toxic exposure in these regions have not been well described[37].

### Perinatal and Neonatal Factors

Adverse birth outcomes are closely linked with low maternal socioeconomic status and compromised maternal health such as malnutrition, anemia, and infections. These adverse outcomes include preterm birth, low birth weight, and intrauterine growth restriction, which are related to neurodevelopment disabilities. In the developing world, scarce obstetrical care and poor management of complicated pregnancies and deliveries confound these maternal risk factors. Little research has been conducted into the etiology and prevention of perinatal complications in these regions[37].

### Poverty

Correlations between disability and age, education, income, ethnicity, living arrangements, and gender cannot determine causality. Not only does disability add to the risk of poverty, but conditions of poverty add to the risk of disability[55]. This statement exemplifies the complex multifactorial relationship between socioeconomic status and ID. Children with developmental disabilities in the developed world are a disadvantaged and often marginilized population. However, for the most part, they receive some form of governmental social assistance and health care. The children with developmental disabilities in developing countries are doubly disadvantaged because not only are they marginilized and often completely neglected, but their low-income countries cannot provide the social assistance and health care required to give them the chance at an improved quality of life. In addition, the low-income environment is the strongest and most consistent predictor of mild ID throughout the world[56]. In developing countries, lack of funds translates directly into lack of parental education and awareness, poor maternal and fetal nutrition, lower immunization rates, lack of antenatal care, and reproductive counseling. All of these factors lead to increased rates of intellectual and developmental disabilities in the developing world.

### Future Directions

There is an important role for international organizations to work with the governments of the developing countries to make care for and prevention of developmental disabilities a priority. For example, Inclusion International, a federation of 200 organizations, advocates for the human rights of people with ID worldwide. They are pressing the United Nations and other donor agencies to include people with disabilities in new developmental programs in low-income countries[49]. Organizations such as these are important because the intellectually disabled lack a strong political voice.

Much of the literature on policies in developed countries focused on the adequacy of existing income support programs, residential and community care facilities, and special health-care needs for those with ID. In most developing countries, income maintenance schemes and even reserved employment schemes have limited applicability where there is no effective labor market. Particularly in lower-income countries, disabled people have to rely to a large extent on informal systems for support[55]. Although proven methods of prevention exist, such as maternal immunization against rubella, iodization of salt, and early screening, they are not properly implemented in the developing world. Therefore, policy efforts in developing countries must remain focused on education, prevention, and rehabilitation.

## HEALTH CARE FOR PERSONS WITH INTELLECTUAL DISABILITY

### Israeli Arabs

The Arabs in Israel are a traditional society currently undergoing a transition period involving modernization and increased exposure to the values and customs of the Jewish population in Israel. Many aspects of their cultural and socioeconomic position in Israel create a position of increased risk for the birth of a child with ID. In addition, these factors also contribute to increased morbidity for these children as the utilization of rehabilitation and health services is diminished compared to the Israeli Jewish population. The picture of this doubly disadvantaged population is not unlike that of the ID community in the developing world.

In the traditionally Eastern family, there is a strong focus on the extended families and the majority of care for a disabled child occurs via informal supports. In contrast, the Western family is smaller and care for a disabled child relies more heavily on formal supports such as established medical care and educational facilities provided by the government. As the Arab population in Israel begins to adopt more modern Israeli values, a shift towards reliance on formal supports is being observed[57]. However, a study of Arab mothers with ID children indicated that the use of informal supports helped reduce marital, parental, and economic stress, whereas formal support had no association with stress levels[58]. Therefore, despite the shift to formal support utilization, it is important to encourage the continued use of traditional informal supports as well.

The Bedouin population, a minority within the Arab minority, currently numbers 170,000 persons[59]. This population is traditionally nomadic, however, the vast majority are now established in permanent residences. The process of urbanization is leading to a breakdown of tribal social culture and a transition to social individualism[59,60]. This transition is causing more Bedouin parents to elect to place their ID children in community or residential care centers[57].

The traditional tribal culture of the Bedouin place marriage and family at the very center. The rate of consanguinity is very high and this population does not usually perform prenatal screening for Down syndrome and other congenital causes of ID. Another risk group is the very young mothers who tend not to use adequate antenatal care, including prenatal screening.

As with the developing world, nutritional sufficiency is a problem in the Arab and Bedouin populations. Of Bedouin children, 60% have iron deficiency anemia, which if left untreated can damage the cognitive performance[37,57]. Again, like the developing world, exposure to toxic substances is higher in the Bedouin community. One study conducted in Beer-Sheva assessed the cognitive and neuropsychological symptoms, relative to matched controls, of children who had been hospitalized for poisoning before the age of 3[61]. More than 90% of the hospitalizations were Bedouin children. The findings showed that kerosene poisoning impaired verbal memory and in addition, the poisoned group demonstrated impaired ability to inhibit prepotent behaviors when distracted[61]. The Arab population of Israel consistently ranks lowest in terms of occupation, income level, and education level[6]. All three of these economic indicators are strong predictors of health and disability. Poverty is the strongest and most consistent predictor of mild ID throughout the world[56].

There are additional concerns when it comes to serving the Arab population in Israel. In this case, it is often the providers who take a more traditionally Western, developed world approach, while the recipients of the services come from a traditionally Eastern, developing world context. When cultural perceptions about the value of health and rehabilitation are divergent, there is an inherent difficulty in service delivery. According to the AAMR definition[11]: “Valid assessment considers cultural and linguistic diversity as well as differences in communication, sensory, motor, and behavioral factors,” which means that in order for assessment to be meaningful, it must take into account the individual’s diversity and unique response factors. The individual’s culture or ethnicity, including language spoken at home, nonverbal communication, and customs that might influence assessment results, must be considered in making a valid assessment.

The issues of cultural sensitivity are relevant to rehabilitation services as well. Most obviously, there are language barriers that exist. But more subtly, the cultural norms differ as well. According to one speech therapist at the Zusman Child Development Center (CDC) in Beer-Sheva that we interviewed, the expectations of knowledge and skills differ greatly between a 3-year-old Jewish girl and a 3-year-old Bedouin girl. Often, only the father in an Arabic family speaks Hebrew, but as the mother is the primary caregiver, there are difficulties eliciting proper information about the child’s behavior; there is even more difficulty ensuring that rehabilitation exercises will be carried out at home as the mother has difficulty understanding the instructions.

At the Soroka University Medical Center in Beer-Sheva, the health and rehabilitation services have a very large and heterogeneous catch-up population serving most of the south of Israel. One study[62] looked at the utilization and the number of appointments for child rehabilitation services by the Bedouin population compared to the general population in the south of Israel at the Zusman CDC. The records of appointments to the CDC between the years 1995–1999 inclusive were studied and randomly chosen to limit the study to January, April, July, and October of each year, and randomly chosen the daily records of nine therapists, three from each discipline (occuptional therapy [OT], physical therapy [PT], and speech and language therapy [SLT]). There were 8,504 appointments during these 4 months of those years, 2,255 of which were for Bedouin and 6,249 for Jewish children. Noncompliance with therapy appointments (NCTA) for the same period for both the Bedouins (31%) and Jewish children (26%) showed a significant difference between the two populations. Of all the Jewish childrens' appointments, the percentage of all three services was similar: 33% to PT, 38% to OT, and 29% to SLT, but for the Bedouin children, the percentage between the three services was significantly different: 62% to PT, 34% to OT, and 3% to SLT. These results seem to indicate that the Bedouin families prefer the PT and OT over the SLT. The results from this study enhanced the need for planning a model to supply health services adapted to clients coming from different cultures. Cultural differences, the accessibility to rehabilitation services, and the economical impact on the family should be considered in order to find a better solution for the patient with special needs.

The situation for the Arab and Bedouin with ID can be seen as a microcosm of the developing world. The unique position of this doubly disadvantaged community relative to the mainstream (Jewish) ID community requires special attention in the form of health policy for ID.

### DISCUSSION

It is the conclusion of this paper that in terms of its policy approach and service delivery to persons with ID, Israel falls somewhere between the developed and the developing world. The philosophical and subsequent policy approaches towards deinstitutionalization and normalization in Israel has, to a certain extent, mirrored changes in the developed world as well as its accompanying problems. However, the increasing incidence of poverty, in addition to the problems faced specifically by the Israeli Arab and Bedouin populations, makes it impossible to situate Israel within the camp of the developed world. Many of the problems faced by the Israeli Arab and Bedouin communities are analogous to problems faced by persons in the developing world.

Israel's unique mediary position enables it to both learn from and instruct the developed and developing world. Problems to be addressed by Israel that parallel difficulties in the developed world include:

- Necessary shifts in public opinion in terms of persons with ID, especially those people working with this population.
- Addressing the needs of doubly disadvantaged groups such as women, the elderly, and ethnic minorities (where issues such as cultural sensitivity are required).
- Poverty issues that exacerbate the risk of disability and that problematize life for persons with developmental disability (in terms of daily living and probability of receiving proper medical care).
- Issues of consanguinity.
- Higher percentage of uneducated mothers.
- Higher degree of cultural difference between health-care providers and health-care recipients.
- Lower levels of prenatal screening.

Medical services for persons with ID have come a long way since the establishment of the modern State of Israel in 1948. Within the framework of the DMR of the Ministry of Social Affairs, medical service is provided at an acceptable level even with the lack of adequate manpower, but there is a lack of relevant services within the community.

We have several wishes and recommendations for the future. We would like to see Israel proceed as Holland did[63], with a subspecialty for family physicians and pediatricians in ID. There is both a need for a raised level of care in the residential care centers, but most certainly also for the community, where today family physicians and pediatricians provide primary care to all the persons with ID living at home or in community-based hostels or protected apartments. In order to facilitate such activities, we would like to have university affiliation where students in medicine and allied professions can be educated to work with persons with ID, to have students on site in residential care centers, and to conduct joint research projects. A step in that direction was taken in 1999 when the National Institute of Child Health and Human Development (NICHD) was established as the research arm of the Office of the Medical Director[64]. This institute is involved in research and education in the fields of child health, disability, ID, and human development with many activities and publications since its establishment.

As previously discussed, many countries around the world have adapted the Scandinavian model of normalization and deinstitutionalization, which developed in the 1970s, where persons were transferred from large state institutions into services in the community. Several states in U.S.[65] have completely closed their institutions and today serve this population in the community. The transfer has not been without complications. In addition to the problems mentioned earlier in the paper, problems such as earlier death of the client, more health problems or lack of health and dental services, and homelessness have been one of the unfortunate by-products of this transition. It is for this reason that we believe that it is not a question of either/or, but instead a combination of both residential care in small institutions and community settings.

We would like to see the future development in Israel move in a direction with a combination of both community care and institutional care for the population of persons with ID[66,67]. We would like to have future institutions for persons with ID established within selected areas around the country in order to provide service for persons unable to stay in the community or at home. The institution should also function as the regional medical center for persons with ID living in the community or at home, since the present model is inadequate. The medical center at the area institution should provide prevention programs, rehabilitation, nursing care, and treatment in the health field, dental health, nutrition, preventive medicine (vaccination, women's health, etc.), physiotherapy, and occupational therapy for the target population within the whole area assigned. It can thus function as the organizational base for persons with ID and their families in order to ensure a high level of service and support integration and coordination. We advocate establishing four regional centers throughout Israel to treat intensive nursing patients as well as to function as a regional multidisciplinary outpatient facility for all persons with ID in the region providing medical, dental, occupational and physiotherapy service, supervision, assessments, education, and research.

Currently in Israel, the Ministry of Social Affairs allocates 10% of the DMR budget to community care and 85% to residential[29]. The time has come for a reallocation of resources and manpower. This reallocation should also lead to the establishment of a Health Division within the Ministry of Social Affairs, as an independent division with its own administration, manpower, and budget affiliated directly to the Minister and the Director General. The new division should be responsible for all medical, health care, and allied professions within the entire Ministry and its government, public, and private facilities working under license or auspices of the Ministry.

So far, the medical service or health care has been fragmented and spread around the Ministry with very little intercommunication and coordination (other than in cases of emergencies). Working as a nurse, physician, or allied professional in the Ministry has not been an attractive position (we have to compete with better and more interesting jobs like intensive care or surgery) and the result has been that the professionals in the various institutions are usually retired professionals, professionals who could not get a position in the traditional medical system in Israel, or professionals who work full time in the traditional system and come to us to work "on the side" and therefore are not really part of "the system". Continuity is very important to the clients and problems we deal with. Most of the medical care the Ministry provides is to people with disabilities, chronic illness, or severe social problems, and it is very important to have a long-term perspective, contact, training, and involvement. If the professionals change all the time because the work (with, for example, ID or disability in general) is without possibilities for advancement, for credit, for training, or for academic affiliation, the clients are the ones who suffer in the end. The reform has to be done from within the Ministry because the traditional medical system is not interested in dealing with care for the disabled. Traditional medicine is interested in emergencies and short-term hospitalizations and not in chronic illness, rehabilitation, or long-term chronic care. The Ministry is dealing with social medicine, but has not applied the model for the work. The establishment of a Health Division will make better management, better utilization of resources, better setting of priorities, better training, and better research and development, where there will be a professional system and environment to work in.

In order to ensure a coordination of services for persons with ID, local area coordinators should be appointed the task of improving local services and ensuring that necessary information is at hand for individuals and caregivers. Their tasks would include long-term planning to support people with ID and to ensure that their current and future needs are sufficiently met. This planning should be focused on the individual and should, in part, take the form of "personal life plans", whereby the coordinator works with the individual and his or her caregiver(s) in order to implement an individualized long-term support plan based on the person and families specialized needs. The plan will take all of the client's health needs into account including dental, ophthalmic, pharmaceutical, hearing, communication, and physiotherapy, as well as any other social support they may require[8]. In order to ensure that the needs of this specialized population are met, policy makers must have detailed and up-to-date information about the demographic needs of the population.

To address the specific needs of the Israeli Arab and Bedouin populations, we suggest the appointment of a special advisor on Arab and Bedouin care within the umbrella of the Health Division for persons with ID. This individual would coordinate with the different service delivery areas to ensure that the needs of this population are being met in a culturally sensitive manner, which includes improving the rates of prenatal screening and antenatal care and allocating more money to mobile health units. The informal supports (due to extended family support) that exist within the Arab community should be accounted for in policy planning. It is our opinion that instead of attempting to break the family support system, the British and Danish model of nurse home visitation could be implemented[59].

### CONCLUSION

It is the position of this paper that future policy for Israel must recognize its unique position. It can learn from the policies formulated by developed regions such as the U.K., Scandinavia, and the U.S., who have been leading the way in terms of policy formulation on ID, yet it must do so in the context of its socioeconomic reality. In particular, the distinct problems faced by the Arab and Bedouin community in terms of ID must be formally accounted for in Israel's future policies. Research from the developing world would be instructive to this end. It was the goal of this paper to present an international literature review on the shift in philosophy, policy, and treatment towards persons with ID with a specific focus on the State of Israel. This global approach led to certain policy recommendations that take into account the uniqueness of Israel's position from a social, economic, religious, and demographic perspective. It is the hope of its writers that this paper will lead to an increased awareness of the challenges faced by persons with ID and their providers in all sectors of Israeli society and that the necessary policy recommendations will ultimately be adopted.

### ACKNOWLEDGMENTS

This review paper was conducted as part of an assignment at a month summer internship in 2004 by Ilana Halperin and Aliza Shupac at the National Institute of Child Health and Human Development affiliated with the Office of the Medical Director, Ministry of Social Affairs, Jerusalem and the Faculty of Health Sciences, Ben Gurion University of the Negev, Beer-Sheva, Israel.

### BIOSKETCHES

**Ilana Halperin**, medical student, Faculty of Medicine and Dentistry, University of Western Ontario, Canada and summer intern in 2004 at the National Institute of Child Health and Human Development in Israel. E-mail: ihalperi@uwo.ca

**Aliza Shupac**, political science student, Faculty of Arts, Department of Political Sciences, McGill University, Montreal, Quebec, Canada and summer intern in 2004 at the National Institute of Child Health and Human Development in Israel. E- mail: ashupac@hotmail.com

**Mohammed Morad, MD**, is a specialist in family medicine, lecturer in family medicine at the National Institute of Child Health and Human Development, Division of Community Health, Ben Gurion University of the Negev, and the medical director of a large area clinic in the city of Beer-Sheva. Publications include those on Bedouin health, health aspects, spiritual health, and aging in persons with intellectual disability, and he is a presenter on topics like health policy and services for disadvantaged at national and international conferences. E-mail: morad62@barak-online.net

**Joav Merrick, MD, DMSc,** is professor of child health and human development affiliated with the Zusman Child Development Center, Division of Pediatrics and Community Health at the Ben Gurion University, Beer-Sheva, Israel; the medical director of the Division for Mental Retardation, Ministry of Social Affairs, Jerusalem; and founder and director of the National Institute of Child Health and Human Development. He has numerous publications in the field of child health and human development, rehabilitation, intellectual disability, disability, health, welfare, abuse, advocacy, quality of life and prevention. Dr. Merrick received the Peter Sabroe Child Award for outstanding work on behalf of Danish Children in 1985 and the International LEGO-Prize ("The Children's Nobel Prize") for an extraordinary contribution towards improvement in child welfare and well being in 1987. E-mail: jmerrick@internet-zahav.net. Website: www.nichd-israel.com
